# *α*-Conotoxin TxIB Improved Behavioral Abnormality and Changed Gene Expression in Zebrafish (*Danio rerio*) Induced by Alcohol Withdrawal

**DOI:** 10.3389/fphar.2022.802917

**Published:** 2022-02-01

**Authors:** Kailin Mao, Xiaodan Li, Zongde Chen, Xiaoqian Dong, Dongting Zhangsun, Xiaopeng Zhu, Sulan Luo

**Affiliations:** ^1^ Key Laboratory of Tropical Biological Resources, Ministry of Education, Hainan University, Haikou, China; ^2^ Medical School, Guangxi University, Nanning, China

**Keywords:** alcohol withdrawal, *α*6*β*2∗ nAChR, *α*-conotoxin TxIB, zebrafish, transcriptome analysis, RT-PCR, ELISA, monoamine neurotransmitters

## Abstract

**Background and Purpose:** Alcohol use disorder (AUD) is a serious public health issue and affects the lives of numerous people. Previous studies have shown a link between nicotinic acetylcholine receptors (nAChR) and alcohol addiction. However, the role of *α*6*β*2* nAChR in alcohol addiction remains obscure, and whether *α*6*β*2* nAChR can be used as a potential drug target for alcohol withdrawal need to be studied.

**Methods:** Zebrafish (*Danio rerio*) were exposed to 0.2% alcohol for 14 days followed by 7 days of repeated withdrawal and then retro-orbitally injected with *α*-conotoxin TxIB (a selective *α*6*β*2* nAChR antagonist). Open Field Test was applied to characterize zebrafish behavior parameters. The monoamine neurotransmitter amounts and their mRNA expression in the zebrafish brain were identified using ELISA and quantitative real-time PCR (RT-PCR). RNA-sequencing (RNA-seq) and subsequent bioinformatics analysis were employed to explore the potential network regulation of TxIB after alcohol withdrawal.

**Results:** The max speed in the center area of the Open Field Test was significantly higher in the withdrawal group whereas TxIB injection corrected this abnormality. The amount and mRNA expression of monoamine neurotransmitters did not change significantly after alcohol withdrawal and TxIB administration. RNA sequencing of zebrafish brain indicated a total of 657 genes showed aberrant expression and among which 225 were reversed after TxIB injection. These reversed genes were significantly enriched in the calcium ion binding pathway and the gene expression profile was further validated by RT-PCR.

**Conclusion:** Our finding suggests *α*-conotoxin TxIB improved behavioral abnormality induced by alcohol-withdrawal, and changed gene expression mainly in the calcium signaling pathway. Therefore, *α*-conotoxin TxIB is expected to become a potential therapeutic agent for alcohol withdrawal.

## Introduction

Alcohol abuse poses a great risk to human physical and mental health and a serious economic burden to society. According to WHO, 3 million people die each year from the inappropriate use of alcohol, a figure that accounts for 5.3% of the overall annual global death toll (World Health Organization, 2019). Alcohol is a predisposing factor for numerous diseases, and approximately 5.1% of diseases are directly or indirectly triggered by alcohol. The damage caused by alcohol is particularly pronounced in younger age groups, with approximately 13.5% of deaths in the 20-39 age range being alcohol-related. Alcohol can cause damage to multiple organs, with damage to the cardiovascular system being widely reported, and a study by Whitman IR et al. in a California population older than 21 years of age showed that alcohol abuse significantly increased the incidence of atrial fibrillation, myocardial infarction, and congestive heart failure. In addition, alcohol abuse greatly increased the risk of heart disease in people without conventional cardiovascular disease risk factors (Whitman et al., 2017). Currently, four medications are approved by FDA to treat the AUD, which are disulfiram, naltrexone (oral and long-acting injectable formulations), and acamprosate. They all have different mechanisms and limitations. Besides, the neural basis in alcohol use disorders is not well understood.

Studies in recent years have shown that nicotinic acetylcholine receptors (nAChRs) in the mesolimbic dopamine system play a crucial role in regulating the rewarding effects of alcohol (Powers et al., 2013). The non-selective nAChR antagonist mecamylamine prevented alcohol-induced cumulative DA levels from increasing when applied to the pre-ventral tegmental area (VTA), suggesting that nAChRs in VTA were involved in the regulation of alcohol reward (Touchette et al., 2018). Besides, the large expression of *α*6 subunit-containing nAChRs in midbrain dopamine neurons indicated that they may be involved in reward-related behaviors (Yang et al., 2009). Another study showed *α*6 nAChR high-expression mice consumed significantly more alcohol than the control group and were more likely to form conditioned place preferences with a low concentration of alcohol (Powers et al., 2013). Studies using patch-clamp recording on the cells confirmed that *α*6* (* represents the presence of other subunits in the nAChR complex) nAChR but not other nAChR subtypes are selectively activated by the low dose alcohol (0.1–5 mM). In addition, alcohol also increased the transient frequency and amplitude of dopamine neurons in the nucleus accumbens slices of mice brain and was further blocked by the *α*6*β*2* nAChR antagonist, *α*-conotoxin MII (Gao et al., 2019). Thus, selective *α*6*β*2* nAChR antagonist is expected to play a significant role in studying the role of *α*6*β*2* nAChR in alcohol abuse and may propose a novel therapeutic strategy.

Previously evidence has shown nAChRs are involved in the withdrawal process. Bhutada PR et al. (Bhutada et al., 2010) reported acute administration of mecamylamine (1–4 mg/kg, intraperitoneally) dose-dependently attenuated ethanol withdrawal-induced signs, and these effects were comparable with those of diazepam (1–2 mg/kg, intraperitoneally). Additionally, chronic administration of mecamylamine into ethanol diet-fed mice markedly attenuated the ethanol withdrawal sign scores, thus supporting the contention that nAChR is involved in ethanol dependence. In animals experiencing withdrawal after chronic ethanol treatment, acute nicotine exposure was sufficient to prevent abstinence symptoms. Similarly, symptoms were prevented when alcohol was injected acutely in mice undergoing nicotine withdrawal (Perez et al., 2015). These experiments provide evidence for the involvement of the nicotinic cholinergic system in alcohol withdrawal. In terms of the specific role of *α*6 nAChR in the withdrawal process, Jackson KJ et al. (Jackson et al., 2009) reported using MII[H9A:l15A], which is a selective *α*6* nAChR antagonist, blocked the nicotine withdrawal-associated conditioned place aversion and anxiety-related behavior in the elevated plus maze. Pang X et al. (Pang et al., 2016) reported selectively blocking *α*6*β*2 nAChR in the medial habenula (MHb) alleviated anxiety in mice undergoing nicotine withdrawal, while *α*6*β*2 nAChR antagonists had little effect on nicotine naïve mice. Together, these results suggest a role for the *α*6 nAChR subunit in the withdrawal process.

Conotoxin is a class of polypeptide toxins found in carnivorous marine mollusks and usually consists of 10–40 amino acids. Previous studies have shown that many conotoxins exhibited unique activity of blocking various ion channels such as nAChRs, sodium ion channels, potassium ion channels, etc (Lebbe et al., 2014). In recent years, several conotoxins that can act on *α*6*β*2* nAChR have been discovered such as MII and ArIB (Everhart et al., 2004; Whiteaker et al., 2007), and these neuropeptides have played an important role in the study of *α*6*β*2 * nAChR-related pathophysiology. Through administering *α*-conotoxin MII locally into the VTA of mice, researchers found that MII significantly reduced alcohol-induced locomotor stimulation and dopamine overflow in the ventral striatum. Besides, MII significantly reduced voluntary alcohol intake in both rats and mice (Larsson et al., 2004). However, most of these peptides have multiple nAChR targets, making it difficult to accurately determine the function of specific receptors. Conotoxin TxIB was found to be the most specific *α*6*β*2* subtype nAChR antagonist with high affinity as it is the only compound that is capable of distinguishing the *α*6*β*2* nAChR from all other subtypes (Luo et al., 2013). Therefore, TxIB is an ideal pharmacological tool in the study of *α*6*β*2* nAChR’s function *in vivo* and *in vitro*. Our lab has previously reported using TxIB as the tool to study its effect on nicotine-induced conditioned place preference (CPP) and found TxIB significantly inhibited the ability of nicotine inducing CPP in mice, additionally, we found monoamine neurotransmitters including dopamine, GABA, and noradrenaline were reduced in nucleus accumbens, hippocampus and prefrontal cortex after TxIB administration (You et al., 2019).

Over the last two decades, zebrafish (*Danio rerio*) has been proven to be a prominent model system in the research of behavioristics and neuroscience (Stewart et al., 2014). Many researchers used zebrafish to study alcohol-induced behavior change. Gerlai R et al. compared the difference of alcohol-induced behavior change among four distinct zebrafish populations (Gerlai et al., 2008). They also detect the neurotransmitter amounts of dopaminergic and serotoninergic systems in the brain of zebrafish exposed to different alcohol concentrations both chronically and acutely (Gerlai et al., 2009). Oliveira TA and others (Oliveira et al., 2013) reported alcohol exposure led to a significant change in the prey-predator relationship and stress axis activation of zebrafish. The function and mechanism of nAChR are conserved across species. Zirger JM et al. (Zirger et al., 2003) cloned three nAChR subtypes (*β*3, *α*2, and *α*7) of zebrafish and found cDNA sequences were highly similar to that of other species. It is also worth noting that nAChR was expressed shortly (several hours) after fertilization. Braida et al. (Braida et al., 2014) applied multiple nAChR agonists and antagonists to evaluate their impacts on the learning and memory of zebrafish, their finding suggests agonists and antagonists that work in rodents or humans also work in zebrafish. Ackerman KM et al. (Ackerman et al., 2009) used *in situ* hybridization to study the location distribution of *α*6 and *α*4 nAChR in the early stage of zebrafish. They found *α*6 nAChR in zebrafish expressed mainly on several parts of the central nervous system, especially in catecholaminergic neurons in the midbrain, which is consistent with rodents and humans. Their finding further indicates zebrafish is an eligible model system to study *α*6 related dopaminergic signaling as well as the brain reward system.

The aim of the current study is two-fold: 1) To identify the behavior change induced by alcohol withdrawal and TxIB administration in zebrafish. 2) To correlate the behavior alterations after chronic alcohol treatment and in response to TxIB injection with gene expression profile. With this aim, we applied behavior recording to detect abnormality induced by alcohol withdrawal. Furthermore, ELISA and RT-PCR were performed to identify monoamine content in the zebrafish brain. Finally, RNA-seq and subsequent bioinformatics analysis were used to identify regulated genes and pathways.

## Materials and Methods

### Zebrafish Housing

Adult zebrafish (wildtype, AB strain) with a gender ratio of 1:1 were obtained from the China zebrafish resource center. The age of the fish was about 4 month-old and their body length was between 3∼4 cm. They were kept in a 60 L tank with water filtered using a reverse osmosis filter system and the water temperature was kept constant at 25 ± 0.5°C. Continuous filtration and ventilating in the tank were provided. The home tank was kept in a room under 14 h light/10 h dark cycles. Zebrafish were fed with the granular tropical fish food twice a day. Besides, all zebrafish were housed for at least 2 weeks in the tank with optimal water quality before the experiment. All the fish were drug-naive and used in a single experiment only. The animal study was reviewed and approved by the Animal Ethics Committee of Hainan University. All procedures were in accordance with ethical standards.

### Alcohol Treatment

Zebrafish were treated with the escalating alcohol concentration to minimize their mortality (Tran et al., 2015). Briefly, zebrafish were first introduced to 0.1% (volume/volume) alcohol-water on the first day, then alcohol concentration was increased by 0.1% every other day, which was 0.2% on the third day. The alcohol concentration reached the top (0.2%) and was held for the next 14 days. From the third week, zebrafish were taken back to the non-alcohol water for 3 h per day to produce an alcohol withdrawal process. On the 21st day, zebrafish were withdrawn from 0.2% alcohol for the last time before the drug injection and the behavior test. The final concentration of 0.2% was determined through a preliminary experiment to insure the alcohol does not cause a substantial number of deaths during the long-term exposure.

### TxIB Synthesis and Potency Identification

All compounds subjected to biological assays were confirmed with >95% purity. Crude linear TxIB was synthesized by GL Biochem (Shanghai, China). Cysteine residues were protected in pairs with either S-trityl on Cys2 and Cys8, or S-acetamidomethyl on Cys3 and Cys16. The purity of crude peptides is greater than 70%. Then the linear TxIB was oxidized to form two disulfide bonds using a two-step oxidation method described previously (Luo et al., 2013). The molecular weight of the folded TxIB was determined using Electrospray Ionisation Mass Spectrometry and the purity was greater than 95%. The HPLC chromatogram and the mass spectra of TxIB were presented in Supplementary Figure S1. The *in-vitro* bioactivity of TxIB was similar on rat and human *α*6/*α*3*β*2*β*3 nAChR (Zhangsun et al., 2017) (a chimera that forms functional *α*6*β*2*-nAChRs *in vitro* (Azam et al., 2008)) and it was confirmed using Voltage-clamp Recording as described previously (Luo et al., 2013).

### Drug Administration and Behavior Test

Lyophilized TxIB was dissolved in normal saline solution (0.9%) and then injected at 10 μL per fish for a final dose of 1 mg/kg using the retro-orbital injection method (Pugach et al., 2009). Briefly, zebrafish were anesthetized in an ice/water bath within 10 s and then removed to a sponge pad for injection. The control and withdrawal groups were injected with 10 μL of normal saline solution per fish to serve as controls. After injection, fish were transferred into freshwater for recovery. Behavior and other test were conducted five hours after injection to ensure fish were recovered from anesthesia. The drug treatments were divided into three groups: control, withdrawal, and withdrawal + TxIB. The Control group indicates the zebrafish without alcohol treatment, the withdrawal group indicates the zebrafish undergoing alcohol treatment, withdrawal + TxIB group indicates the withdrawal zebrafish with TxIB injection.

Behavior tests were performed during the daytime from 10:00 a.m. to 5:00 p.m. to eliminate the influence of biorhythm. Fish were gently moved from the home tank to the behavior testing apparatus followed by video recording. The apparatus of open field for zebrafish referred to a previous study (Kyzar et al., 2012). Briefly, a transparent cylinder (diameter: 20 cm) was filled with water up to 15 cm, horizontally divided into two concentric circles (center and periphery). The wall of the cylinder was covered by white sheets to prevent fish from seeing their surroundings. After the 2-min adaption period, individual fish was recorded for 5 min *via* the camera positioned above the cylinder. After recording, zebrafish was moved to its home tank. Only system water was used during the open field test. Time spent in and the number of entries into the center area were monitored as indexes of anxiety-like behavior. The total distance traveled and the velocity of the movement was recorded as parameters of locomotion. Video files of each trial were analyzed using automated video-tracking software: Smart 3.0 (Panlab Harvard Apparatus, Spain). It allows the recording of trajectories, global activity and performs the calculations of a wide range of analysis parameters including the speed, total distance, etc.

### Quantification of Neurotransmitters

Following five hours after drug injection, zebrafish were anesthetized by immersion in an ice-water mixture and decapitated immediately for whole-brain tissue sampling. Each brain tissue was stored individually in a microcentrifuge tube and put into liquid nitrogen for quick-freezing. Afterward, the brain sample was suspended with 15 µL/mg ice-cold PBS buffer followed by homogenization using a tissue homogenizer. Then the sample was centrifuged at 3,000 rpm for 20 min and the supernatant was transferred to a new microcentrifuge tube for ELISA assay. The amount of dopamine, serotonin, noradrenaline and gamma-aminobutyric acid was analyzed using corresponding commercial Enzyme-Linked Immunosorbent Assay (ELISA) kits (X-Y Biotechnology, Shanghai) according to the manufacturer’s protocol. Total protein content was measured using the BCA protein quantification kit (X-Y Biotechnology, Shanghai) and the quantity of each neurotransmitter was standardized per mg of protein.

### RNA-Sequencing and RT-PCR

Zebrafish were treated identically to the behavior test. Following treatment, zebrafish were anesthetized by immersion into the ice-water bath. After cutting the head instantly from the fish body, the brain was dissected from the head and rinsed in the cold PBS solution. The brain tissue was then put into a freezing tube and transferred into the liquid nitrogen immediately. Ten brains were pooled together for one biological replicate and the total RNA was extracted using FastPure Cell/Tissue Total RNA Isolation Kit (Vazyme Biotech co., Ltd.) following the manufacturer’s instructions. The purity, concentration, and integrity of RNA samples were identified to guarantee the quality of samples for transcriptome sequencing. Afterward, library construction was conducted with the following processes: The mRNA was enriched with magnetic beads with Oligo (dT), and randomly interrupted by adding Fragmentation Buffer, then the first cDNA strand was synthesized with six-base random primers (random hexamers) using mRNA as the template, then dNTPs, RNase H and DNA polymerase I were added to synthesize the second cDNA strand and the cDNA was purified using AMPure XP beads. The purified double-stranded cDNA was then terminally repaired, A-tailed, and ligated to sequencing junctions, followed by fragment size selection using AMPure XP beads. Finally, the cDNA library was enriched by PCR. After the library construction, the effective concentration of the library (>2 nM) was accurately quantified using the qPCR method to ensure the quality of the library. Then the high-throughput RNA sequencing was performed on an Illumina NovaSeq 6000 sequencer at Biomarker Biotechnology Co., Ltd. (Bei Jing). The sequencing read length was 150 bp paired-end. The clean data of each sample reached 5.79 Gb, and the Q30 base percentage was 93.22% and above. Subsequent bioinformatics analysis including differentially expressed genes analysis was performed using BMKCloud (www.biocloud.net). Clean Reads of each sample were aligned with the designated reference genome respectively (GRCz11). EdgeR package was used for differential expression analysis and fold change ≥2 and *p*-value < 0.05 were used as the screening criteria. The RNA-seq raw data has been deposited in the Gene Expression Omnibus - NCBI (https://www.ncbi.nlm.nih.gov/geo/) with the accession number: GSE186926.

For RT-PCR analysis, total RNA of the whole zebrafish brain was extracted using FastPure® Cell/Tissue Total RNA Isolation Kit (Vazyme Biotech Co., Ltd., China). The concentration of RNA was measured by Nanodrop Spectrophotometer (Nanodrop Technologies) and the integrality was verified by electrophoresis in an agarose gel. Afterward, 1 μg total RNA per sample was reverse transcribed into cDNA using HiScript® II Q Select RT SuperMix (Vazyme Biotech Co., Ltd., China). RT-PCR analysis was then performed in the qTOWER 3 Real-Time PCR Thermal Cycler (Jena Analytical Instruments GmbH, Germany) using ChamQ SYBR qPCR Master Mix (Vazyme Biotech Co., Ltd., China). At least three independent biological replicates were examined in the current study, with each being examined in duplicate. The mRNA expression level was calculated using Double Delta Ct Analysis and presented as relative fold-change of the control group after normalization to the *rpl13a* mRNA levels. *Rpl13a* has been investigated to be the most reliable reference gene in the previous study (Baronio et al., 2018). Primer sequences were listed in Table 1.

**TABLE 1:** List of the primers for RT-PCR analysis.

Gene symbols	Full gene name	Forward primer (5'∼3')	Reverse primer (5'∼3')
*th1*	Tyrosine hydroxylase 1	GAC​GGA​AGA​TGA​TCG​GAG​ACA	CCG​CCA​TGT​TCC​GAT​TTC​T
*th2*	Tyrosine hydroxylase 2	CTC​CAG​AAG​AGA​ATG​CCA​CAT​G	ACG​TTC​ACT​CTC​CAG​CTG​AGT​G
*dbh*	Dopamine beta-hydroxylase	TGC​AAC​CAG​TCC​ACA​GCG​CA	GCT​GTC​CGC​TCG​CAC​CTC​TG
tph1a	Tryptophan hydroxylase 1a	TTC​AAG​GAC​AAT​GTC​TAT​CG	GGG​AGT​CGC​AGT​GTT​TGA​TG
tph1b	Tryptophan hydroxylase 1b	AGC​CAA​ATG​TAG​AAT​GCG​TGA​A	CCA​CAA​TGT​CCA​GCT​CGT​GTC
tph2	Tryptophan hydroxylase 2	CCA​GGA​GTG​CCT​CAT​TAC​CA	TAA​GTC​GAT​GCT​CTG​CGT​GT
*cacng1a*	Calcium channel, voltage-dependent, gamma subunit 1a	CAT​GGG​AAA​GCT​GCA​TGG​AC	GGT​TGC​CCT​CAT​CTC​TTC​AC
*tpma*	Alpha-tropomyosin	CCA​CGC​TCT​CAA​CGA​CAT​GA	CGA​TGG​AGA​AAA​GCG​GCA​AC
*cacna1sb*	Calcium channel, voltage-dependent, L type, alpha 1S subunit, b	CAG​CAG​TAT​CTC​CCT​CGC​TG	ACG​ATG​TCT​GCG​TAA​GCC​AA
*atp2a1l*	ATPase sarcoplasmic/endoplasmic reticulum Ca2+ transporting 1, like	TTC​GCT​CTT​TCT​GTC​AGT​CCA​A	GTA​GGC​CAG​ACA​TTC​AGC​CG
*actc1b*	Actin alpha cardiac muscle 1b	AGT​ACT​CCG​TCT​GGA​TCG​GT	GTA​CGG​CTG​AGA​GAC​TGA​GAG
*trdn*	Triadin	GCT​ATC​GCC​ATT​GCT​CTT​GG	TGC​GAT​ACC​AGC​AGT​GCT​TA
*myl4*	Myosin, light chain 4, alkali	CCA​ACA​TGT​GTC​CAG​GTC​CA	ATC​AAA​CAC​TCG​CAA​GCC​CT
*atp2a1*	ATPase sarcoplasmic/endoplasmic reticulum Ca2+ transporting 1	TGG​GCT​GCA​CAT​CTG​TCA​TT	CAA​ATT​GGC​CGC​ACT​TGA​CA
*cox6a2*	Cytochrome c oxidase subunit 6A2	TGA​CCG​ACC​CCC​TTT​TGA​AG	AAC​GTT​TGC​CAT​GCA​GAC​AC
*atp1a1a.4*	ATPase Na+/K+ transporting subunit alpha 1a, tandem duplicate 4	ATA​CGG​GAC​TGA​CCT​GAC​CA	AGG​ATG​GCA​CCA​ATC​CAC​AG
*rpl13a*	Ribosomal protein L13a	AGA​GAA​AGC​GCA​TGG​TTG​TCC	GCC​TGG​TAC​TTC​CAG​CCA​ACT​T

### Data Analysis

Data were visualized and analyzed using Graphpad Prism (version 8.3). All data were expressed as a mean ± SEM (standard error of the mean) of at least three independent experiments. One-way ANOVA followed by a Tukey test was performed to determine the statistical significance between groups. *p* < 0.05 was considered to be statistically significant (*p < 0.05, **p < 0.01, ***p < 0.001, ****p < 0.0001). Heatmap (Figure 4B) was generated using TBtools: An Integrative Toolkit Developed for Interactive Analyses of Big Biological Data. Gene Ontology enrichment was conducted using The Database for Annotation, Visualization, and Integrated Discovery (DAVID), and the figure (Figure 4C) was generated using ggplot2 (R package). Venn diagram (Figure 4A) and the figure of KEGG pathway enrichment (Figure 4D) were generated using BMKCloud (www.biocloud.net). KEGG map (Figure 8) was generated using KEGG Mapper (https://www.genome.jp/kegg/mapper/).

## Results

### Behavior Test

**FIGURE 1:**
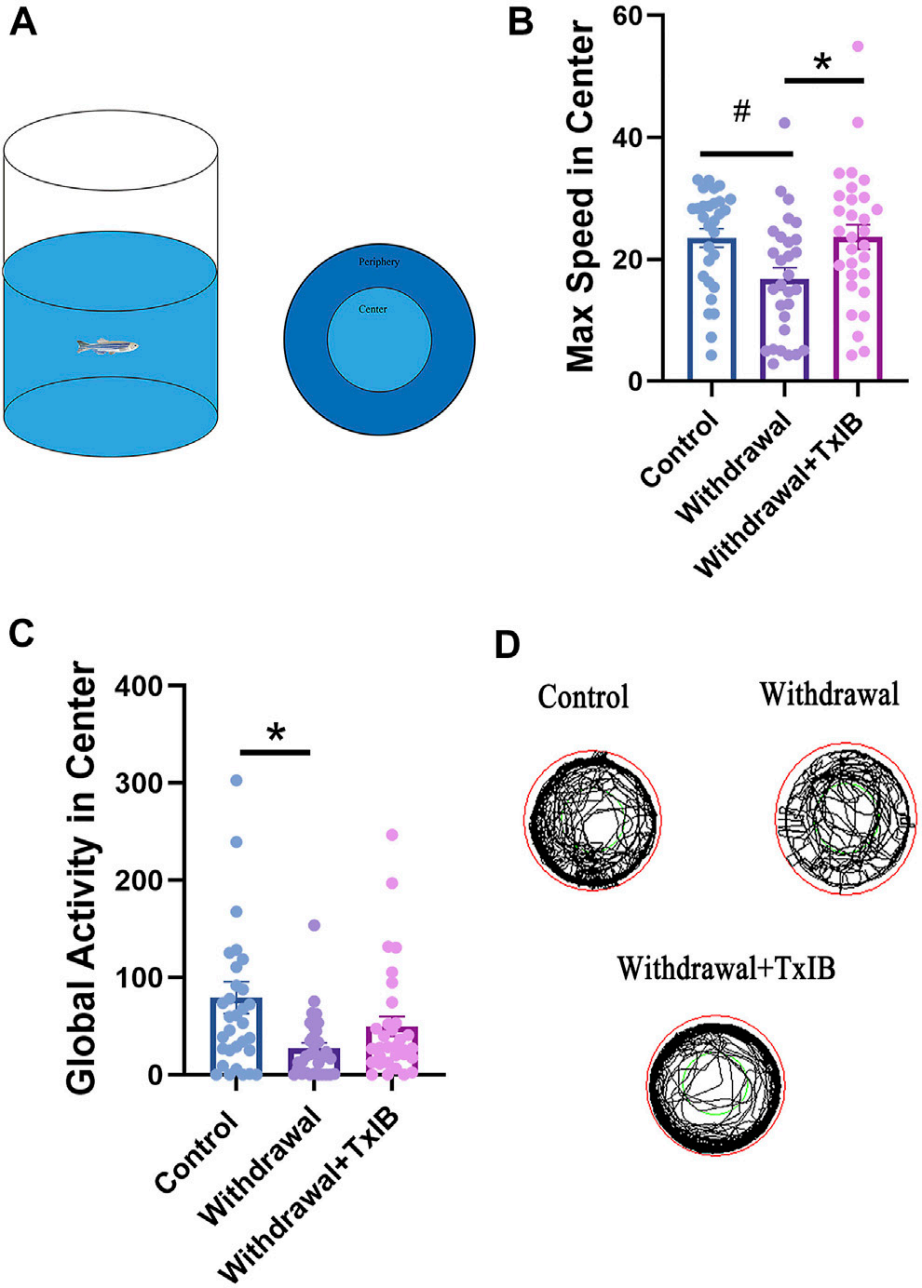
The behavior profile of zebrafish in the open field test. Lyophilized TxIB was dissolved in normal saline solution (0.9%) and then injected at 10 μL per fish for a final concentration of 1 mg/kg using the retro-orbital injection method. The control and withdrawal groups were injected with 10 μL of normal saline solution per fish to serve as controls. **(A)** The diagram of the open field test with individual fish, the tank was divided into two equal virtual zones, center and periphery; **(B)** The max speed in the center zone; **(C)** The global activity in the center zone; **(D)** Representative trajectory chart of individual fish in the open field apparatus. Data represented as mean ± SEM (30 zebrafish per group). * indicates *p* < 0.05, # indicates *p* is between 0.05 and 0.1 (one-way ANOVA followed by Tukey’s multiple comparisons test was performed).

To assess if TxIB can exert any effect on the locomotion or motor pattern of withdrawal zebrafish, an open field test Figure 1A was conducted with 30 zebrafish per treatment group. As shown in Figure 1B, the max speed in the center zone of the TxIB group (23.67 ± 2.01 cm/s, *p* = 0.04) was significantly higher than the withdrawal group (16.81 ± 1.82 cm/s, *p* = 0.04) and it was close to the level of the control group (23.50 ± 1.52 cm/s). In line with the max speed, the withdrawal group showed significantly lower global activity in the center zone (27.03 ± 5.66 cm^2^/s, *p* = .01) than the control group (79.45 ± 16.53 cm^2^/s, *p* = .01) whereas the TxIB group was slightly higher (49.58 ± 10.16 cm^2^/s, *p* = .52). Figure 1C. Figure 1D showed the representative trajectory chart in the open field test.

### Monoamine Neurotransmitter Amounts in the Zebrafish Brain

**FIGURE 2:**
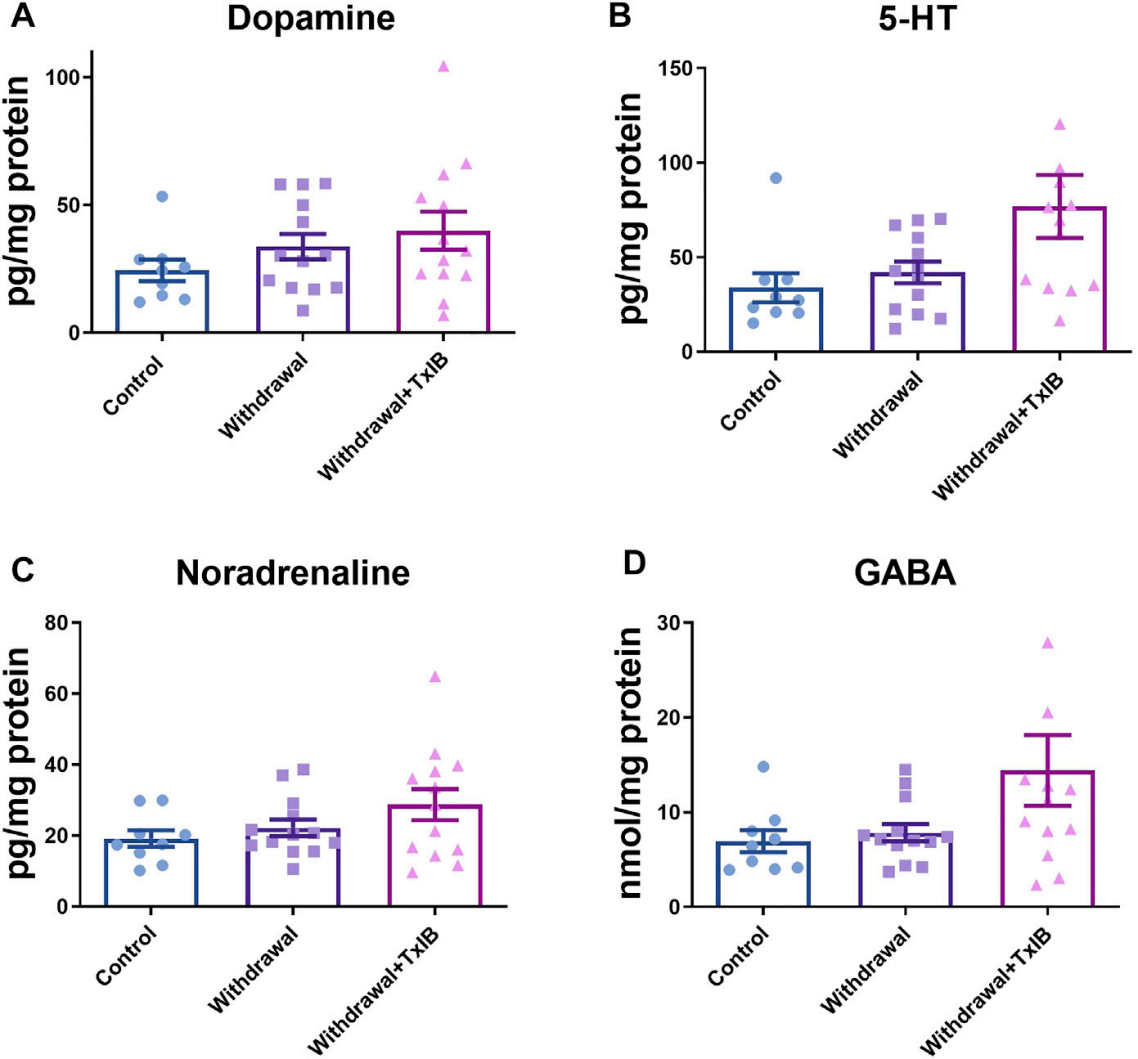
Monoamine neurotransmitter content in the whole zebrafish brain; **(A)** Dopamine content; **(B)** 5-HT content; **(C)** Noradrenaline content; **(D)** GABA content. TxIB concentration of 1 mg/kg was used. Data are mean ± SEM of 9–13 zebrafish per group. One-way ANOVA followed by Tukey’s multiple comparisons test was performed.

To assess whether alcohol withdrawal treatment and TxIB injection can alter monoamine neurotransmitters in the zebrafish brain, neurotransmitter amounts of dopamine, serotonin, noradrenaline, and GABA were measured using ELISA assay (Figures 2A–D). These neurotransmitters of the TxIB group were slightly higher than the normal and withdrawal group, whereas the ordinary one-way ANOVA test found the difference did not reach statistical difference (*p* > 0.05). The dopamine level in the normal group reached 24.50 ± 4.22 pg/mg protein, which was the lowest among treatments. The dopamine level of withdrawal and TxIB group were slightly higher and reached 33.74 ± 4.94 pg/mg protein, 40.03 ± 7.41 pg/mg protein respectively. The amount of serotonin was similar to dopamine. In the normal group, the serotonin was 33.88 ± 7.70 pg/mg protein, and in the withdrawal and TxIB groups, it was marginally higher. As for noradrenaline, the normal group (19.18 ± 2.33 pg/mg protein) was approximately the same as the withdrawal group (22.17 ± 2.31 pg/mg protein), whereas the TxIB group (28.77 ± 4.40 pg/mg protein) was slightly higher. The GABA content of the three groups has a similar pattern as noradrenaline. The normal group (6.94 ± 1.16 nmol/mg protein) was almost equal to the withdrawal group (7.84 ± 0.92 nmol/mg protein) and the TxIB group (14.43 ± 3.74 nmol/mg protein) was slightly higher.

### The mRNA Expression Level of Monoamine Neurotransmitter Synthetase in the Zebrafish Brain

**FIGURE 3:**
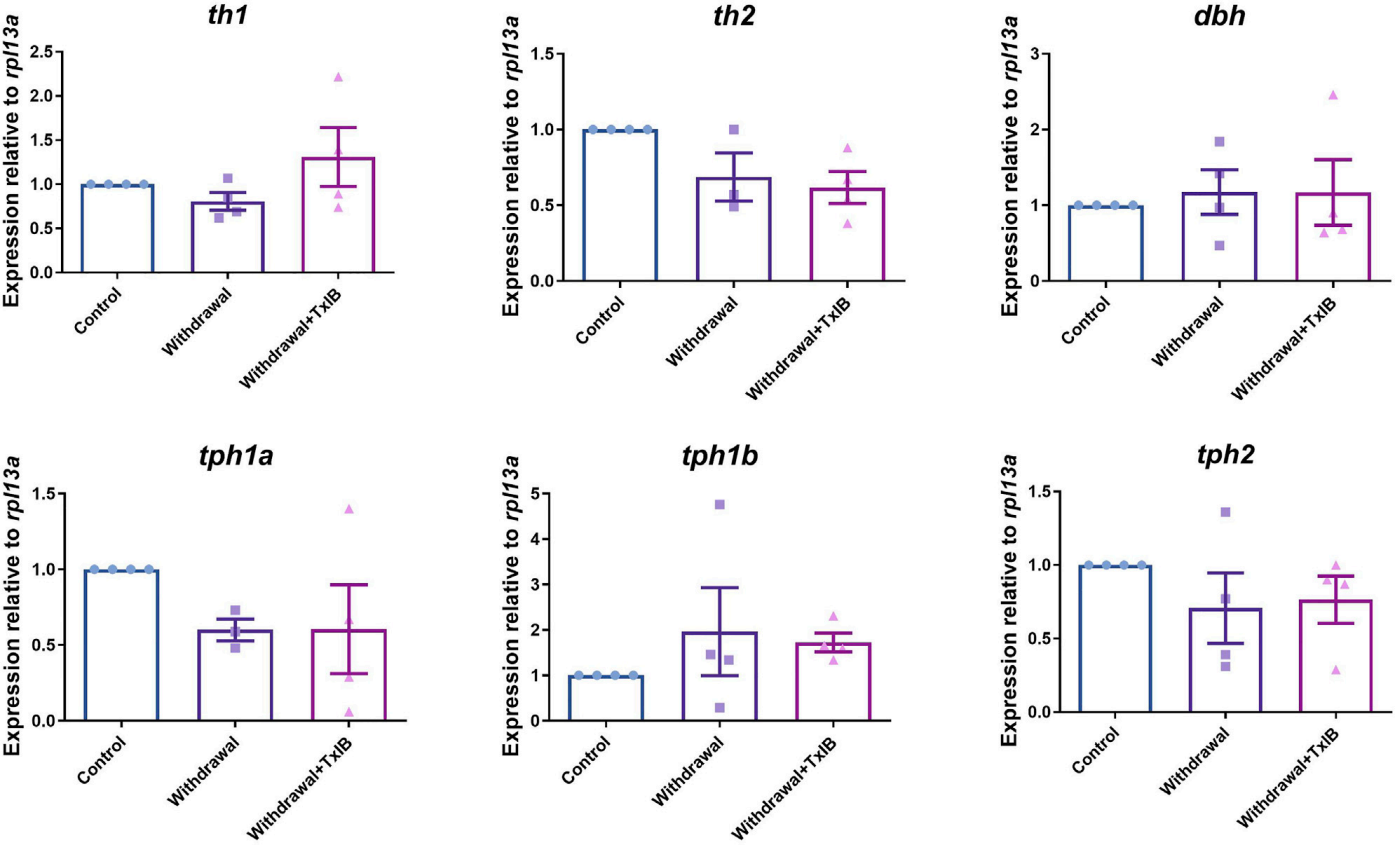
mRNA expression of rate-limiting enzymes in the synthesis of monoamine neurotransmitters, *th1*, *th2* are two isoforms of the rate-limiting enzyme of dopamine synthesis, *dbh* is the rate-limiting enzyme of noradrenaline synthesis, *tph1a*, *tph1b*, and *tph2* are three isoforms of the rate-limiting enzyme of 5-HT synthesis. TxIB concentration of 1 mg/kg was used. Data are mean ± SEM of 4 zebrafish per group. One-way ANOVA followed by Tukey’s multiple comparisons test was performed.

We assessed the mRNA expression level of the rate-limiting enzymes in monoamine neurotransmitters (dopamine, serotonin, noradrenaline) synthesis. Six genes were chosen including *th1, th2*, *dbh*, *tph1a*, *tph1b*, and *tph2* as *th1*, *th2* are two isoforms of the rate-limiting enzyme of dopamine synthesis, dbh is the rate-limiting enzyme of noradrenaline synthesis, *tph1a*, *tph1b*, and *tph2* are three isoforms of the rate-limiting enzyme of 5-HT synthesis. The primer sequences used here were shown in Table 1. As a result, the expression level of these genes was not altered significantly by alcohol withdrawal or TxIB treatment in the whole zebrafish brain (Figure 3).

### TxIB Regulated Calcium Signaling Pathway in Zebrafish Brain

**FIGURE 4:**
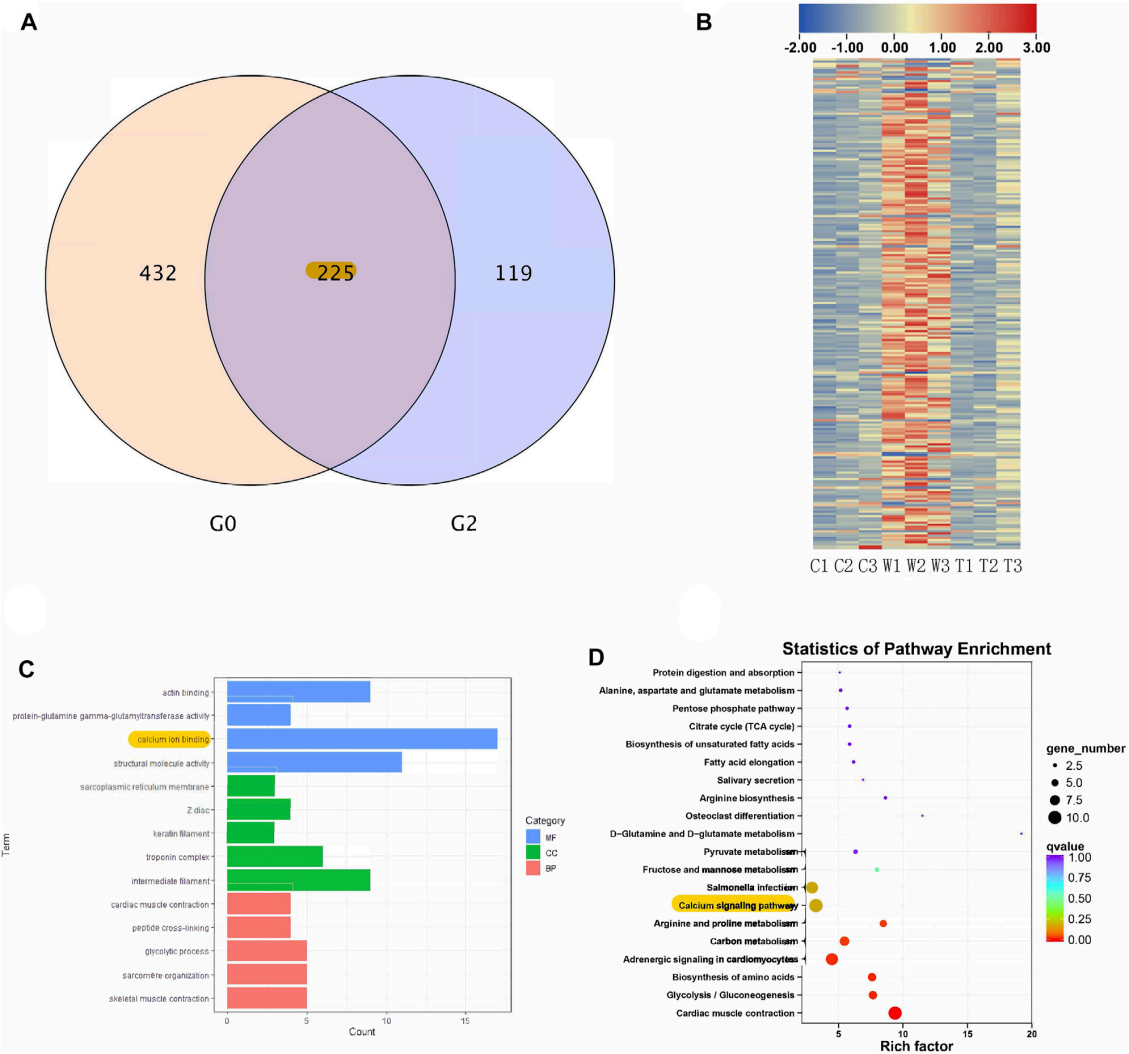
RNA sequencing results; **(A)** Venn diagram of overlapped genes between control vs withdrawal (G0) and withdrawal vs withdrawal + TxIB (G2) datasets; **(B)** Heatmap of expression of overlapped 225 genes (“C” indicates Control group, “W” indicates alcohol withdrawal group, “T” indicates withdrawal + TxIB group); **(C)** Gene Ontology enrichment of overlapped 225 genes. MF: molecular function CC: cellular component BP: biological process; **(D)** KEGG pathway enrichment of overlapped 225 genes.

RNA sequencing was used to identify genes and signaling pathways that were impacted by alcohol withdrawal and TxIB administration. Between the control and withdrawal groups, 657 genes were detected as differentially expressed, and 344 genes differed in expression between the withdrawal and TxIB groups. Venn diagram showed among these differentially expressed genes (DEG), 225 genes appeared in both groups. These genes were our main focus as their expression pattern was regulated by alcohol withdrawal and further reversed by TxIB (Figure 4A). The heatmap of these 225 genes showed that they were mainly up-regulated (shown as red) by the alcohol withdrawal treatment while in the TxIB group they were down-regulated and recovered to the normal expression pattern (Figure 4B).

**FIGURE 5:**
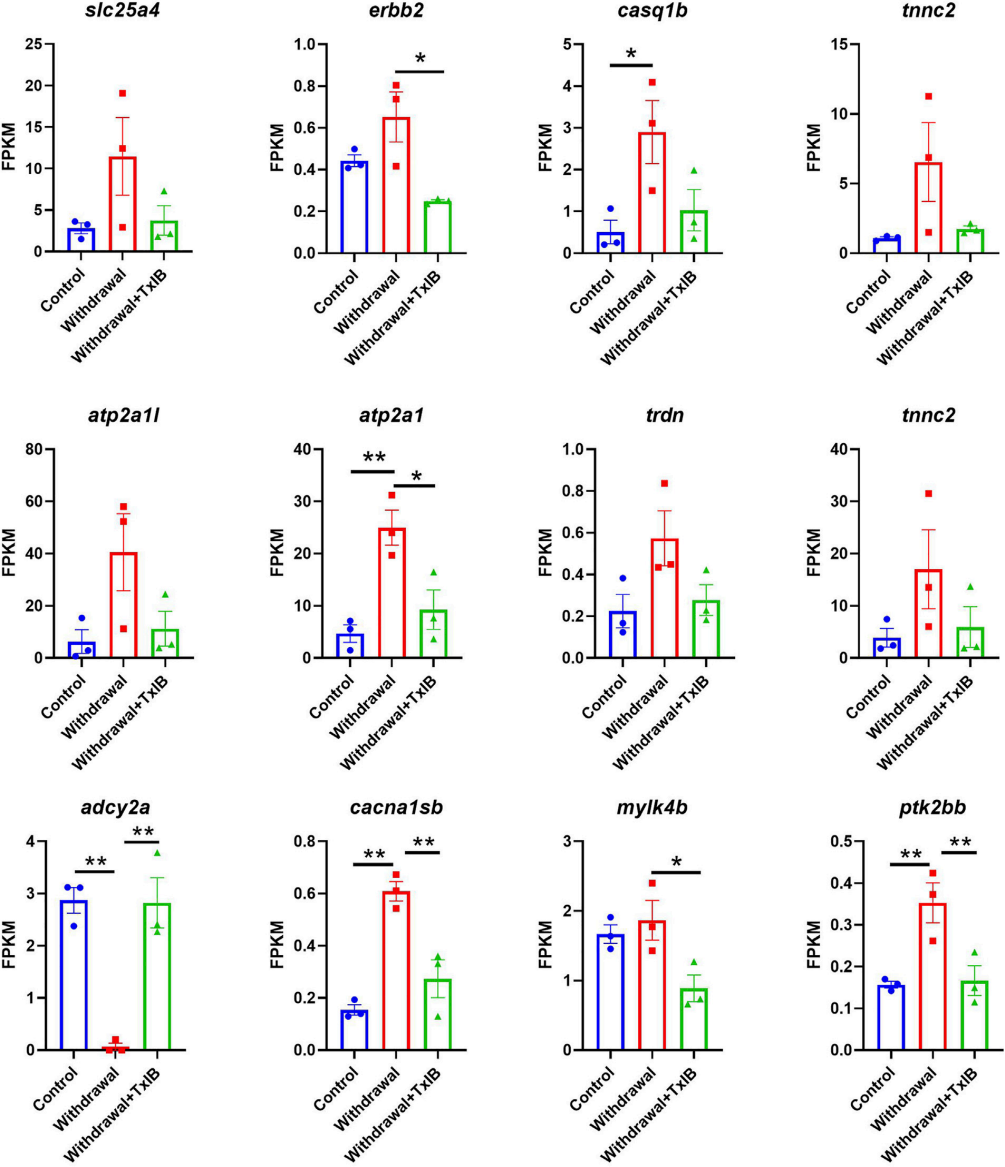
The relative expression profile of genes enriched in the calcium signaling pathway. Data are mean ± SEM of 3 samples per group. * indicates *p* < 0.05, ** indicates *p* < 0.01 (one-way ANOVA followed by Tukey’s multiple comparisons test).

KEGG and GO enrichment analysis showed among all DEGs, genes in calcium ion binding and calcium signaling pathways were significantly enriched, as shown in Figures 4C,D. We further picked out the genes enriched in the calcium signaling pathway from RNA-seq results, there are a total of 12 genes enriched and their relative expression profile was shown in Figure 5. These genes were mainly upregulated by alcohol withdrawal, and down-regulated by TxIB. Among them, *erbb2, atp2a1, cacna1sb, mylk4b, ptk2bb* were significantly down-regulated by TxIB, and *adcy2a* was substantially up-regulated by TxIB administration.

### Protein-Protein Interaction (PPI) Analysis, Module and Hub Gene Screening

To identify the global protein-protein interaction (PPI) network of the DEGs, the DEGs list was input into the STRING database (https://string-db.org/). Supplementary Figure S2 shows the results of PPI network construction in which 176 nodes were identified together with 621 edges (regulations). The average node degree is 7.06 and the PPI enrichment *p*-value is <1.0 e-16. The densest area of interactions appeared around *ttna*, *ttnb*, and *myl1*.

**FIGURE 6:**
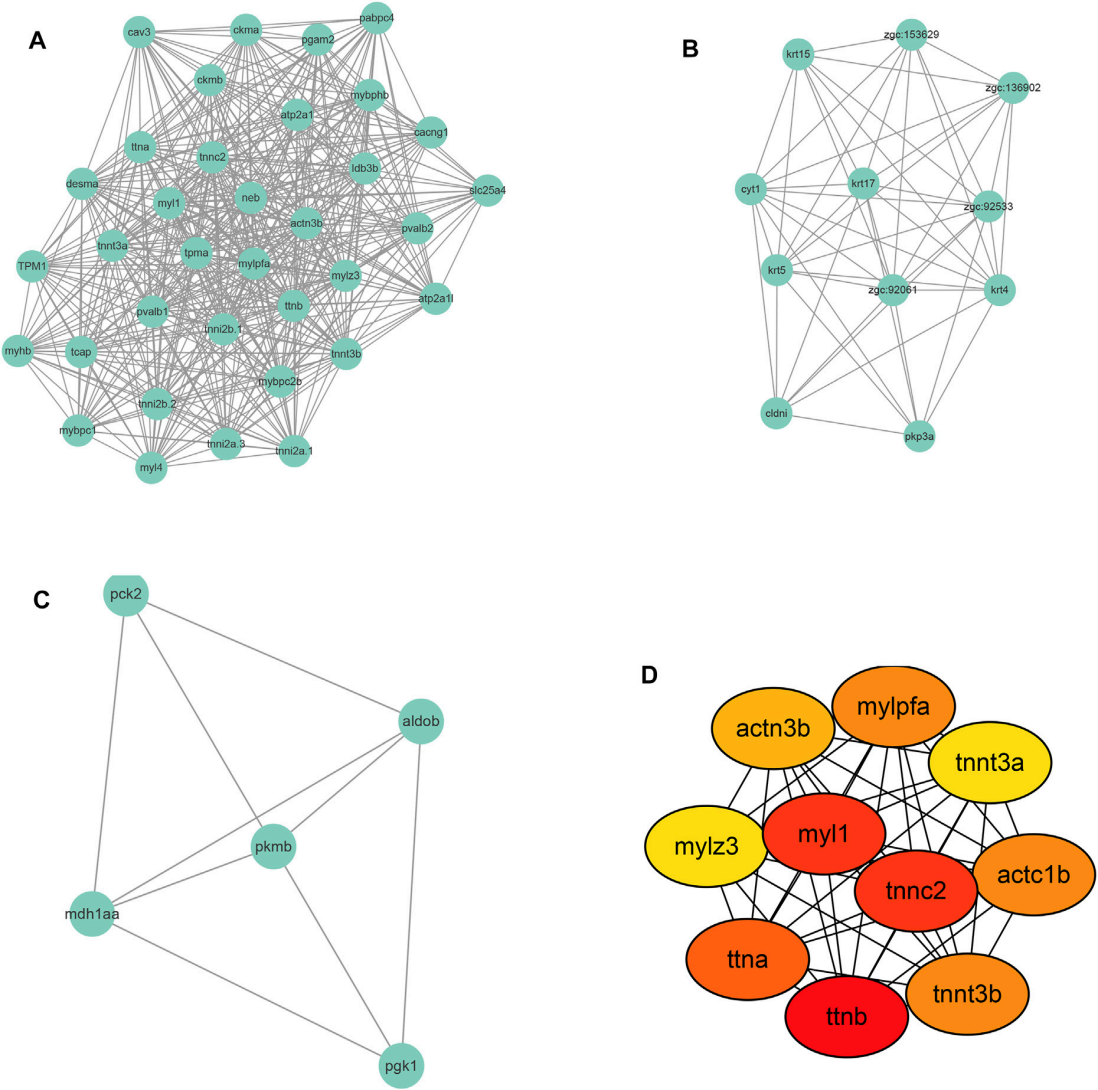
Module screening **(A–C)** and hub gene screening **(D)** from Protein-protein interaction network.

Following PPI network construction, we further employed the MCODE (molecular complex detection) plug-in of Cytoscape software to identify densely connected regions in the PPI network. Figures 6A–C showed the top 3 modules calculated by MCODE. There are 35 nodes and 472 edges in cluster 1 with an overall score of 27.76 and its core is tpma. In cluster 2 there are 11 nodes and 49 edges with the core of cyt1 and the overall score of 9.8, and 5 nodes and 9 edges were found in cluster 3 with mdh1aa as the core and the overall score is 4.5.

Further, the top 10 Hub genes were identified using Cytohubba plug-in in Cytoscape software (Figure 6D). The full name and description of these hub genes were searched in The Zebrafish Information Network (ZFIN) (https://zfin.org/) and listed in Supplementary Table S1. As shown in Supplementary Table S1, the 10 hub genes were mainly involved in the regulation of muscle contraction. As to the Human ortholog(s) of these genes, they were primarily indicated in intrinsic cardiomyopathy and myopathy.

### RT-PCR Validation

**FIGURE 7:**
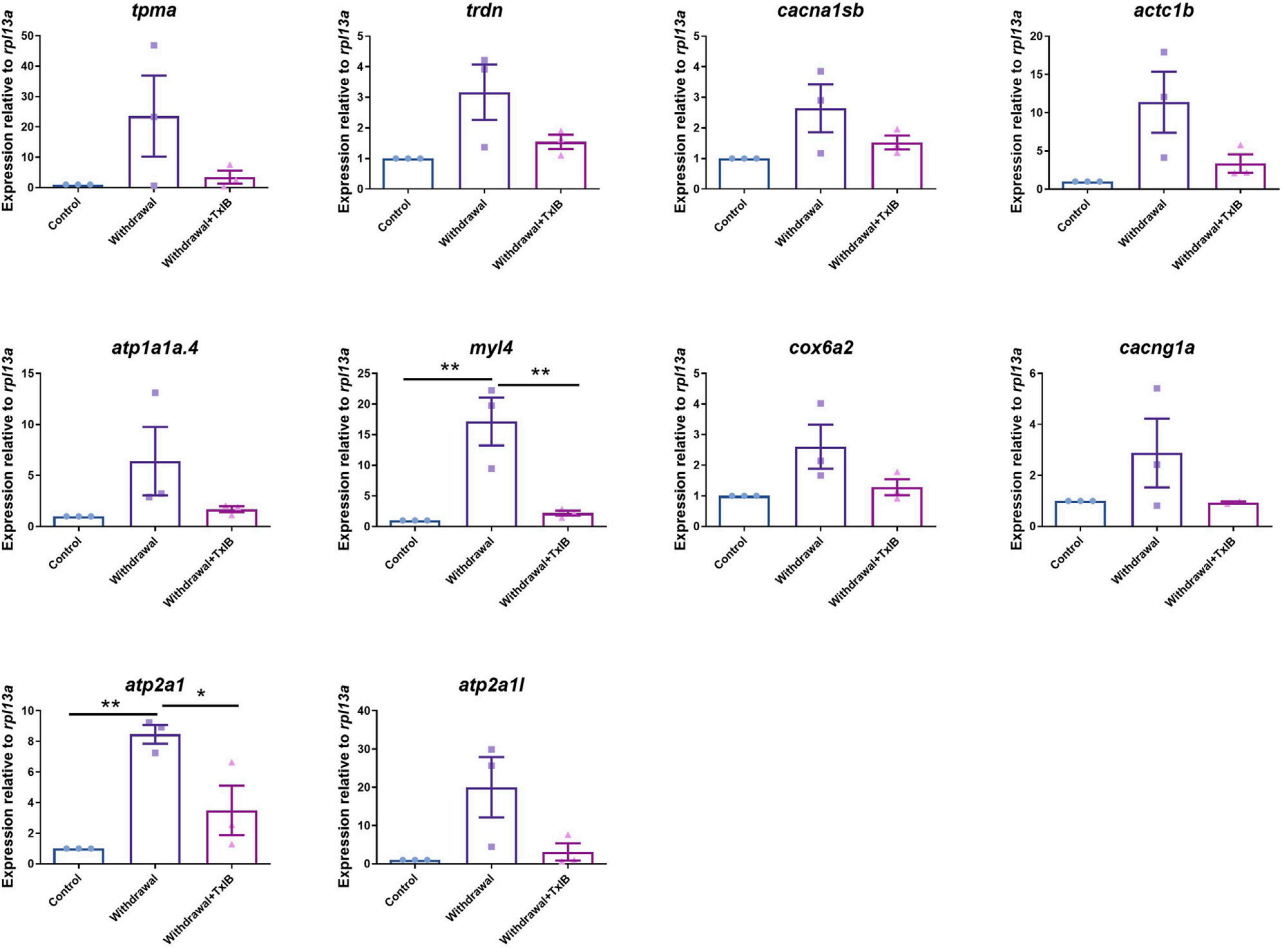
The expression level of genes in calcium signaling and cardiac muscle contraction pathway using RT-PCR. TxIB concentration of 1 mg/kg was used. Data are mean ± SEM of 3 samples per group. * indicates *p* < 0.05, ** indicates *p* < 0.01 (one-way ANOVA followed by Tukey’s multiple comparisons test).

To validate the gene expression profile of RNA-seq results, 10 genes enriched in the calcium signaling pathway and cardiac muscle contraction pathway (*tpma, trdn, cacna1sb, atc1b, atp1a1a.4, myl4, cox6a2, cacng1a, atp2a1, atp2a1l*) were chosen to be validated through RT-PCR. The results (Figure 7) were generally consistent with RNA sequencing data that withdrawal from alcohol increased the expression of these genes while TxIB administration suppressed the expression. In particular, *myl4* expression was elevated up to 16 fold by alcohol withdrawal and reduced to normal status in the TxIB group. *Atp2a1* was upregulated 9 fold in the withdrawal group and the TxIB group was reduced to 5 fold compared to the control group. The rest of the genes all showed an upregulation trend by the withdrawal treatment whereas downregulated by TxIB administration.

## Discussion

This study aimed to elucidate the potential effects of *α*-conotoxin TxIB on alcohol withdrawal zebrafish at behavioral, molecular, and transcriptomic levels. There have been studies focusing on the role nAChR played in alcohol and nicotine addiction. Two reviews have made a profound summary of studies investigating that nAChR was involved in alcohol and nicotine addiction (Feduccia et al., 2012; Rahman et al., 2015). Among numerous subtypes of nAChR, *α*6 nAChR is particularly relevant to alcohol addiction. Using transgenic mice of hypersensitive mutant *α*6 nAChR (*α*6L9′S mice), Powers MS et al. (Powers et al., 2013) found that all the transgenic mice consumed significantly more alcohol than the control group in the Drinking in the Dark test. Besides, a low dose of alcohol (0.5 g/kg) can lead to alcohol-induced place preference in *α*6L9′S mice whereas it was ineffective in control mice. Another strong piece of evidence is produced by Gao F and others (Gao et al., 2019). They constructed a heterologous expression system of human *α*6* nAChR based on SH-EP1 human epithelial cells. Using the whole-cell patch-clamp recording technique, they found the low concentration of alcohol (0.1–5 mM) can significantly enhance *α*6* nAChR mediated currents and this modulation is ineffective on other nAChR subtypes. Furthermore, the frequency and amplitude in nucleus accumbens slices of mouse brain were also enhanced by 5 mM alcohol and further blocked by *α*6*β*2* nAChR antagonist MII. Srisontiyakul J and others (Srisontiyakul et al., 2016) reported a novel small molecule compound—bPiDI which selectively target and block *α*6*β*2* nAChR. Using inbred alcohol-preferring rats, they found 3 mg/kg bPiDI markedly reduced the alcohol self-administration and it had no effect on sucrose self-administration and locomotion of rats. All of these studies have shown that *α*6*β*2* nAChR is a selective target of alcohol and plays a crucial role in alcohol and nicotine addiction. Therefore, compounds that act on *α*6*β*2* nAChR might serve as potential therapeutic agents to alleviate and treat alcohol and nicotine use disorders.

In the open field test, we found that the max speed in the center zone was altered by the withdrawal treatment and further reversed by TxIB. However, the difference in total distance traveled between groups was slight (data not shown). The global activity in the center zone of the withdrawal group was significantly lower than the control group, indicating an increased degree of anxiety after alcohol withdrawal. This is consistent with several studies reporting anxiety-like symptoms after alcohol withdrawal (Krook et al., 2019; Mocelin et al., 2019). However, the global activity in the center zone was not altered significantly by TxIB administration in withdrawal zebrafish. Supplementary Figure S3 showed a behavior test on control fish injected with TxIB. The results also showed the max speed of control fish was not altered significantly by TxIB administration, however, the global activity in the center decreased significantly when control fish were injected with 1 mg/kg TxIB. The results indicate TxIB injection in control fish leads to reduced general locomotion in the center area of the open field. This finding is consistent with a prior study (Kamens et al., 2017) showing *α*6*β*2* nicotinic receptors are involved in locomotor activity. TxIB increased the max speed in withdrawal fish but not in control fish, which might suggest *α*6*β*2* nAChR is activated in alcohol withdrawal, and blocking *α*6*β*2* nAChR by TxIB attenuated this effect in withdrawal fish.

In this study, we did not observe the marked change of monoamine neurotransmitter amount after alcohol withdrawal in the whole zebrafish brain. However, several studies indicated the content of several neurotransmitters was altered after alcohol withdrawal in the specific brain region. Rossetti ZL and others (Rossetti et al., 1992) reported that dopamine displayed a significant fall in the ventral striatum of mice after 6 days alcohol administration and withdrawal. The reason for this discrepancy is possible because the dopamine amount alteration happened in certain brain regions whereas in the whole brain it is hard to detect. Therefore the ventral striatum should be focused on in future studies. However, due to the small size of the zebrafish brain, it is difficult to distinguish and extract different brain regions and it will be easier to operate in animals with larger brain sizes. Laine TPJ et al. (Laine et al., 1999) reported the dopamine transporter binding efficiency decreased substantially in prolonged heavy drinkers and recovered after 4 weeks of alcohol withdrawal. Using zebrafish as the model, Alexandre MCM et al. (Alexandre et al., 2019) found dopamine levels increased significantly after 9 days following 3 week’s intermittent weekly ethanol exposure. However, our study indicated dopamine level only increased slightly after 2 week’s alcohol treatment and 1 week’s repeated withdrawal. This difference might be derived from the different protocols of alcohol exposure. It is also worth noting that the total brain was used to measure dopamine level in Alexandre’s study, therefore it is more comparable with ours. Besides, noradrenaline level in Alexandre’s study showed no marked change after intermittent weekly ethanol exposure, which is consistent with ours.

The monoamine level assay of TxIB on control fish was shown in Supplementary Figure S4, the result showed although dopamine and noradrenaline level were not altered significantly in control fish injected with TxIB, the 5-HT level increased significantly in the (control + TxIB) group, which indicating TxIB is capable of increasing the outflow of 5-HT in the whole zebrafish brain. This result is consistent with our previous experiment showing TxIB increased 5-HT level significantly in male withdrawal zebrafish (Supplementary Figure S5). Therefore, the behavior phenotype observed in the open field test might be induced through a 5-HT level increase after TxIB injection. The 5-HT increase induced by TxIB might be due to its action directly or indirectly on the 5-HT receptor *via* blocking *α*6*β*2* nAChRs.

It is reported that acute alcohol exposure increased dopamine and its metabolite dihydroxy-phenyl acetic acid in the whole brain of zebrafish (Tran et al., 2017). Besides, the protein expression of tyrosine hydroxylase, which is the rate-limiting enzyme of dopamine synthesis, was also up-regulated after acute exposure to 1% alcohol persisting 60 min. Our study showed that the expression of tyrosine hydroxylase mRNA in the whole zebrafish brain did not change significantly between the control and alcohol withdrawal groups. It might be because, after 14 days of alcohol exposure and 7 days of repeated withdrawal, the regulatory mechanism of the dopamine pathway in the zebrafish brain has been inactivated. Besides, 0.2% alcohol used in our study may not be high enough to induce such alteration in dopamine synthesis. However, further study is needed to identify whether 0.2% alcohol acute exposure can induce dopamine regulation in the zebrafish brain.

Alcohol withdrawal influences cardiac function, which was reported by Kähkönen S et al. (Kähkönen et al., 2011). The cardiovascular system of patients during alcohol withdrawal syndrome underwent significant changes characterizing the decrease of heart rate, systolic blood pressure, diastolic blood pressures, and total peripheral resistance. These cardiovascular symptoms were related to the altered central and peripheral adrenergic nerve activity. In patients during early withdrawal, noradrenaline and corticotrophin-releasing hormone concentrations correlated positively with diastolic blood pressure in cerebrospinal fluid (Hawley et al., 1994). In the current study, TxIB ameliorated the gene expression abnormality induced by alcohol withdrawal in the cardiac muscle contraction pathway as shown in Figure 7, indicating its potential to regulate the cardiovascular system in the context of alcohol withdrawal.

**FIGURE 8:**
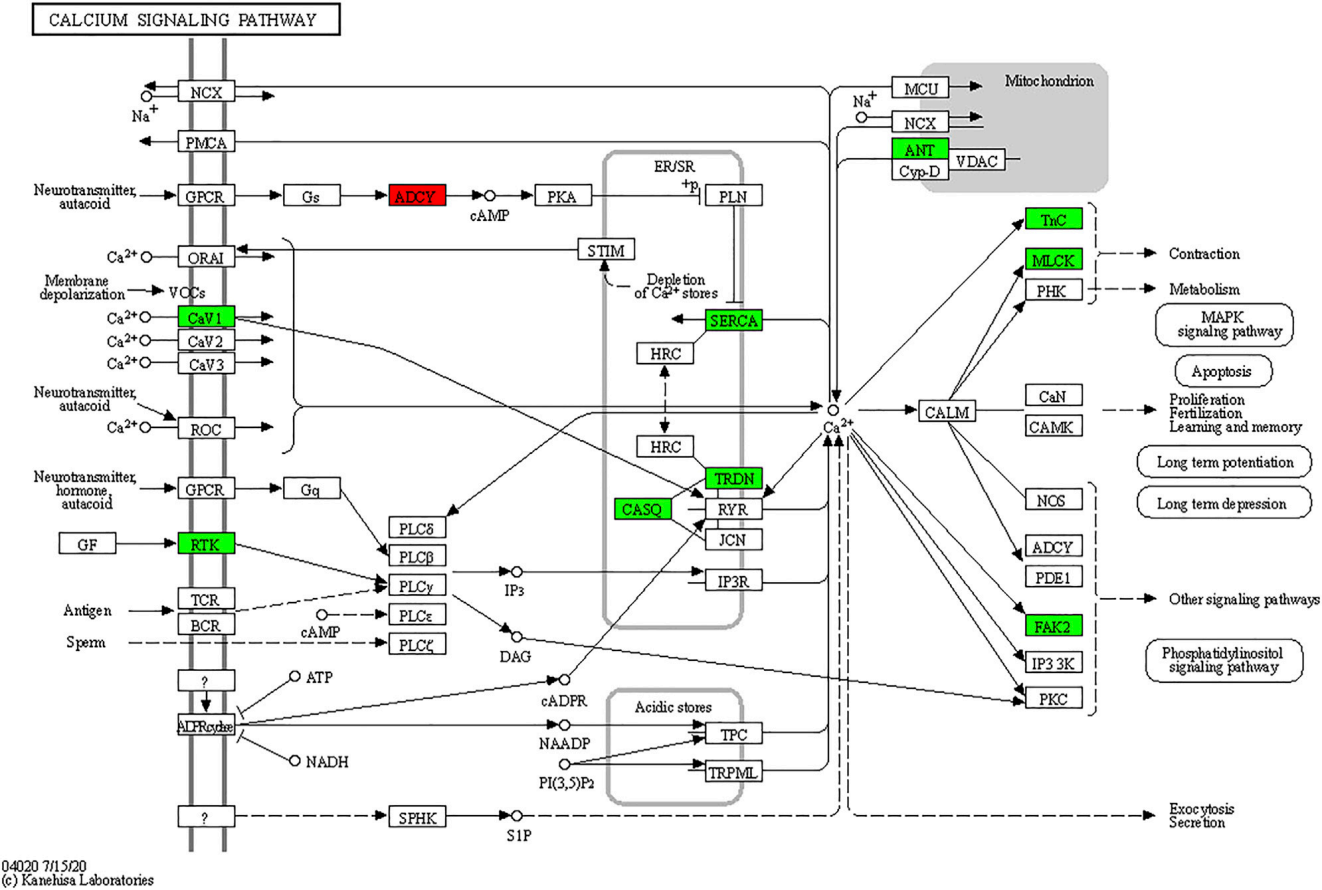
KEGG map of the calcium signaling pathway. The red and green boxes indicate the up-regulated and down-regulated proteins through TxIB administration.

As shown in Figure 8, TxIB administration strongly regulates the calcium signaling pathway, and a total of 10 proteins in the calcium signaling pathway were affected, among them, ADCY was upregulated, Cav1, RTK, SERCA, TRDN, CASQ, ANT, Tnc, MLCK, FAK2 were down-regulated. Evidence has shown that the calcium signaling pathway plays a significant role in the alcohol withdrawal process. Acamprosate (Campral—calcium-bis (N-acetylhomotaurinate)) is currently used as a first-line drug to treat alcohol withdrawal symptoms, Spanagel R et al. (Spanagel et al., 2014) reported acamprosate produces its anti-relapse effects *via* calcium. They found the N-acetylhomotaurinate itself is not capable of inducing any anti-withdrawal effects, however, it is the calcium salt of acamprosate that induces psychoactivity. They also reported that patients with high plasma calcium levels due to acamprosate treatment showed better primary efficacy parameters such as time to relapse and cumulative abstinence. Calcium influx plays important role in alcohol-withdrawal-induced seizures. Newton J et al. (Newton et al., 2021) reported using pharmacologic blocker (SN-6) to prevent calcium from flowing into neurons, which suppressed the occurrence of withdrawal-induced seizures in adult Sprague–Dawley rats, SN-6 also markedly reduced the seizure severity. Calcium ions are essential to many nervous system functions. Voltage-sensitive calcium channels (VSCCs) allow calcium to enter the neuron and can be blocked by dihydropyridines. Researchers found after chronic alcohol intake, the binding sites of dihydropyridines on VSCCs increased dramatically by 50%, and were modulated by the calcium influx into the neuron. Additionally, dihydropyridines as the calcium channel antagonists can alleviate the alcohol-withdrawal induced hyperexcitability in hippocampus slices from rodents undergoing alcohol withdrawal. Administration of Nitrendipine and nimodipine (two dihydropyridines) at a very low dose showed effects of reducing alcohol withdrawal-induced convulsions (Finn and Crabbe, 1997). These results indicate strongly that targeting the calcium signaling pathway can modulate alcohol withdrawal symptoms. Table 2 listed the Top 10 genes that were regulated significantly by TxIB administration. These genes were mainly involved in the calcium-binding process, which is consistent with KEGG enrichment, indicating TxIB injection regulated the calcium-binding process in the alcohol-withdrawal zebrafish.

**TABLE 2:** Top 10 genes that were significantly regulated by TxIB administration.

Number	Gene ID	Gene symbol	Full name	Function	Expression level (mean FPKM^a^)		
					Control	Withdrawal	withdrawal + TxIB
1	ZDB-GENE-000210-33	*nme2b.2*	NME/NM23 nucleoside diphosphate kinase 2b, tandem duplicate 2	Enables nucleoside diphosphate kinase activity. Acts upstream of or within GTP biosynthetic process	36.95 ± 14.71	131.84 ± 41.49	46.11 ± 21.36
2	ZDB-GENE-000322-1	*actc1b*	Actin alpha cardiac muscle 1b	Predicted to be part of dynactin complex	22.18 ± 8.47	109.29 ± 34.26	30.66 ± 11.71
3	ZDB-GENE-040426-2128	*ckmb*	Creatine kinase, muscle b	Predicted to enable creatine kinase activity	20.28 ± 12.00	106.72 ± 32.28	33.64 ± 16.91
4	ZDB-GENE-030115-1	*gapdh*	Glyceraldehyde-3-phosphate dehydrogenase	Predicted to enable glyceraldehyde-3-phosphate dehydrogenase (NAD+) (phosphorylating) activity; microtubule binding activity; and peptidyl-cysteine S-nitrosylase activity	20.09 ± 7.48	80.51 ± 23.26	34.99 ± 14.67
5	ZDB-GENE-000322-4	*pvalb2*	Parvalbumin 2	Predicted to enable calcium ion binding activity	3.51 ± 2.35	61.77 ± 32.96	11.10 ± 9.14
6	ZDB-GENE-000322-6	*mylz3*	Myosin, light polypeptide 3, skeletal muscle	Predicted to enable calcium ion binding activity	9.52 ± 2.64	54.68 ± 20.91	16.41 ± 6.51
7	ZDB-GENE-030520-2	*tnnt3b*	Troponin T type 3b (skeletal, fast)	Predicted to enable tropomyosin binding activity; troponin C binding activity; and troponin I binding activity	9.10 ± 4.48	51.94 ± 20.70	14.81 ± 7.80
8	ZDB-GENE-040801-9	*tnni2b.2*	Troponin I type 2b (skeletal, fast), tandem duplicate 2	Predicted to be involved in cardiac muscle contraction and skeletal muscle contraction	2.43 ± 1.39	42.47 ± 21.87	8.62 ± 5.27
9	ZDB-GENE-081124-1	*myhb*	Myosin, heavy chain b	Predicted to enable ATP binding activity; actin filament binding activity; and cytoskeletal motor activity	1.83 ± 1.01	39.55 ± 10.42	5.19 ± 2.18
10	ZDB-GENE-980526-109	*ckma*	Creatine kinase, muscle a	Predicted to enable creatine kinase activity	9.39 ± 5.57	47.02 ± 16.20	12.60 ± 5.33

a FPKM, stands for Fragments Per Kilobase of transcript per Million mapped reads.

To conclude, our study showed that the specific *α*6*β*2* nAChR antagonist TxIB can alleviate the behavioral abnormality induced by alcohol withdrawal in zebrafish. Transcriptome analysis of zebrafish brain tissue indicated that the expression profile of a total of 657 genes was altered by alcohol withdrawal, among which 225 genes were recovered by TxIB injection. These genes were mainly enriched in calcium signaling pathways. The results of RNA-seq were further validated by RT-PCR. From PPI network construction, 10 hub genes were identified and these genes were primarily myosin and actin coding genes. Besides, the amount and mRNA expression of monoamine neurotransmitters including dopamine, serotonin, noradrenaline, and GABA were not significantly altered in the zebrafish whole brain between treatments. Our findings showed *α*-conotoxin TxIB improved behavioral abnormality induced by alcohol-withdrawal and altered gene expression in the calcium signaling pathway in the zebrafish model. Therefore TxIB would be a potential drug candidate to treat alcohol withdrawal syndrome.

## Data Availability

The datasets presented in this study can be found in online repositories. The names of the repository/repositories and accession number(s) can be found below: https://www.ncbi.nlm.nih.gov/geo/query/acc.cgi?acc=GSE186926.

## Abbreviations

5-HT, serotonin; AUD, alcohol use disorders; BP, biological process; CC, Cellular Component; CPP, conditioned place preference; DA, dopamine; DEG, differentially expressed genes; GABA, gamma-aminobutyric acid; GO, Gene Ontology; KEGG, Kyoto Encyclopedia of Genes and Genomes; MF, Molecular Function; nAChR, nicotinic acetylcholine receptor; PPI, Protein-protein interaction; RNA-seq, RNA-sequencing; RT-PCR, quantitative real-time PCR; VTA, ventral tegmental area.
