# Supplementary material for: α-Conotoxin TxIB Improved Behavioral Abnormality and Changed Gene Expression in Zebrafish (Danio rerio) Induced by Alcohol Withdrawal
Source: Front Pharmacol. 2022 Feb 1;13:802917. doi: 10.3389/fphar.2022.802917 (PMC8844014; doi:10.3389/fphar.2022.802917)
Supplement: Supplementary file 1 [file DataSheet2.docx]

The raw data of RNA-seq in our article has been deposited in Gene Expression Omnibus - NCBI, the link is: <https://www.ncbi.nlm.nih.gov/geo/query/acc.cgi?acc=GSE186926>. The GEO accession number is GSE186926.

Best regards,

Kailin Mao
