# Supplementary material for: α-Conotoxin TxIB Improved Behavioral Abnormality and Changed Gene Expression in Zebrafish (Danio rerio) Induced by Alcohol Withdrawal
Source: Front Pharmacol. 2022 Feb 1;13:802917. doi: 10.3389/fphar.2022.802917 (PMC8844014; doi:10.3389/fphar.2022.802917)
Supplement: Supplementary file 2 [file DataSheet1.docx]

Supplementary Material

# Supplementary Figures and Tables

## Supplementary Figures


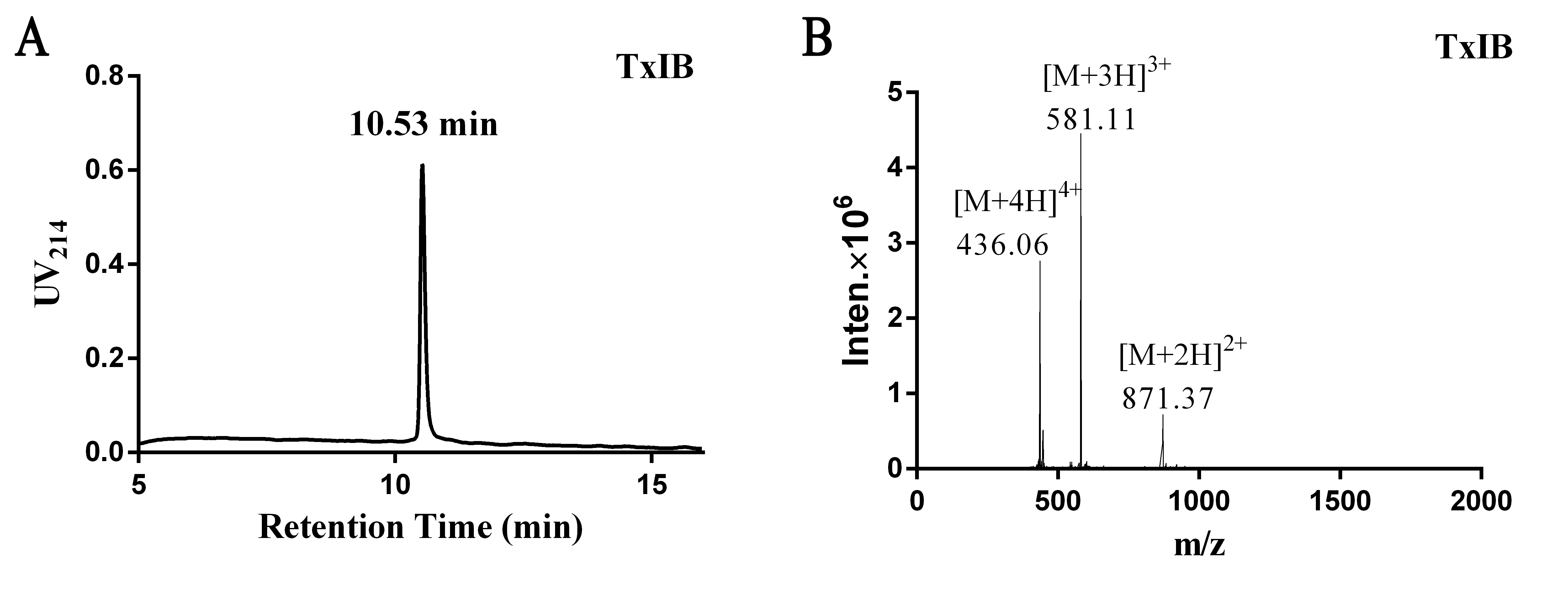


**Supplementary Figure 1.** The HPLC and ESI-MS profiles of α-conotoxin TxIB. The α-conotoxin TxIB were analyzed on a reverse-phase analytical C18 column (5 μm, 4.6 mm × 250 mm) with flow rate 1.0 ml/min. The solvent gradient was from 5% buffer B to 40% buffer B in 20 min where buffer A = 0.1% trifluoroacetic acid (TFA), buffer B = 0.1% TFA and 90% acetonitrile. The absorbance was monitored at 214 nm. (A) The HPLC chromatogram of fully oxidized peptide TxIB; (B) Electrospray Ionisation Mass Spectrometry data for TxIB with an observed monoisotopic mass of 1740.33 Da.


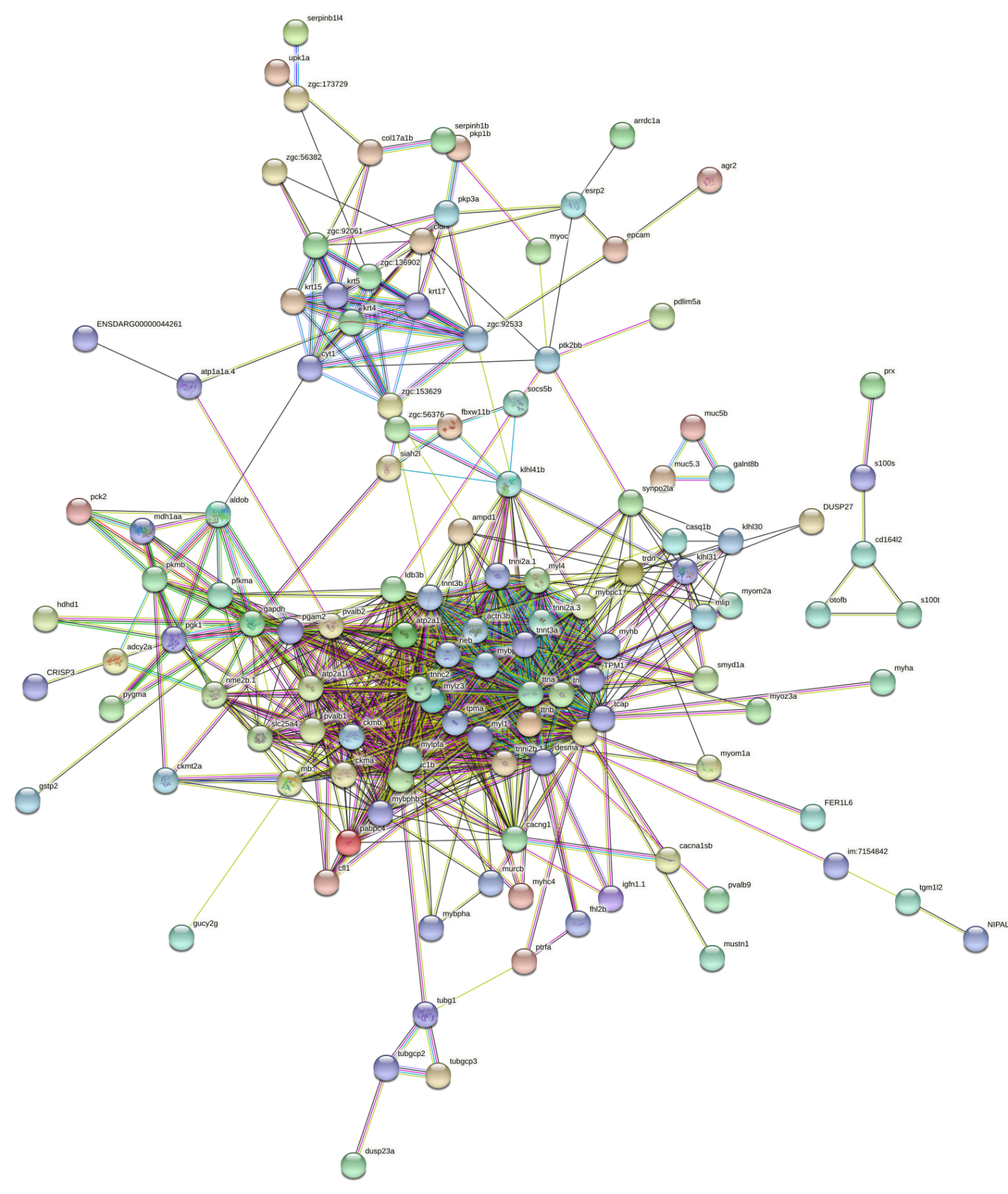


**Supplementary Figure 2**. Protein-protein interaction network from STRING database. Nodes indicate proteins while edges signify the associations between two proteins. Cyan edges indicate known interactions from curated databases whereas rose-red edges signify the interaction that has been experimentally determined. Green, red and blue edges represent the predicted interactions from gene neighborhood, gene fusions, and gene co-occurrence respectively. Yellow, black and light-blue edges indicate interactions predicted from text mining, co-expression, and protein homology respectively. The known or predicted 3D structure of the proteins was shown in the node.


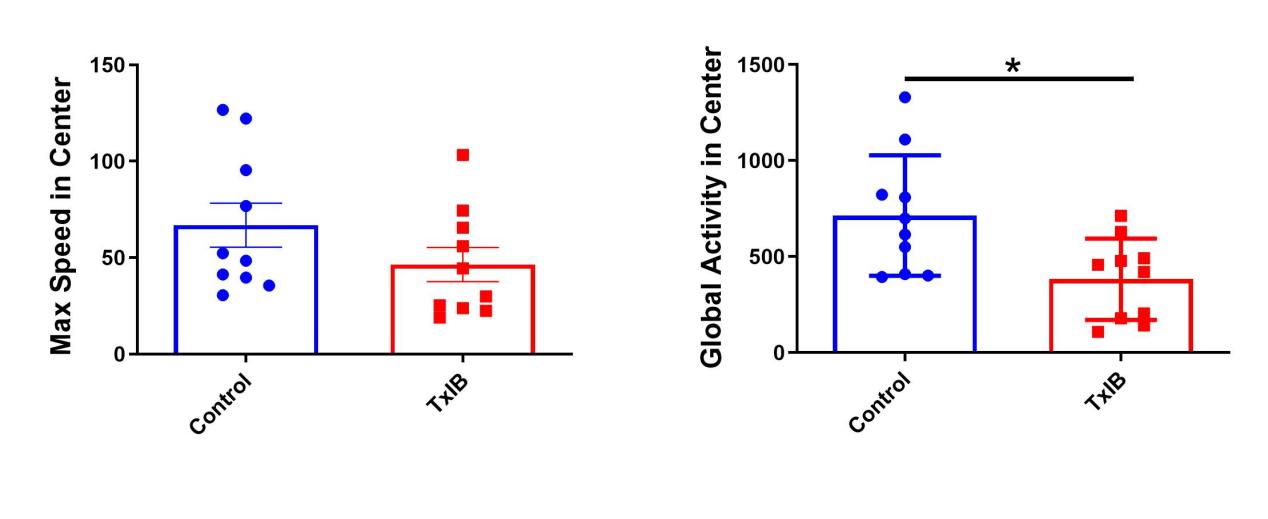


**Supplementary Figure 3**. Open field test. Lyophilized TxIB was dissolved in normal saline solution (0.9%) and then injected at 10 μL per fish for a final concentration of 1 mg/kg using the retro-orbital injection method. The Control group was injected with 10 μL of normal saline solution per fish. Data represented as mean ± SEM (10 zebrafish per group). * indicates *P* < 0.05 (one-way ANOVA followed by Tukey's multiple comparisons test was performed).


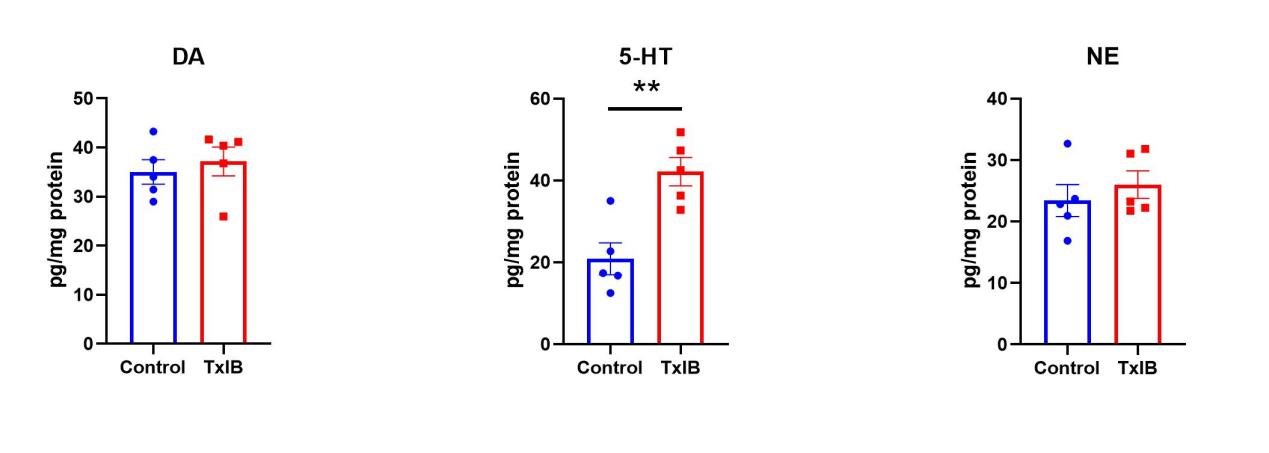


**Supplementary Figure 4**. Monoamine neurotransmitter content in the whole zebrafish brain. TxIB concentration of 1 mg/kg was used. Data are mean ± SEM of 5 zebrafish per group. **indicates *P* < 0.01 (One-way ANOVA followed by Tukey's multiple comparisons test was performed).


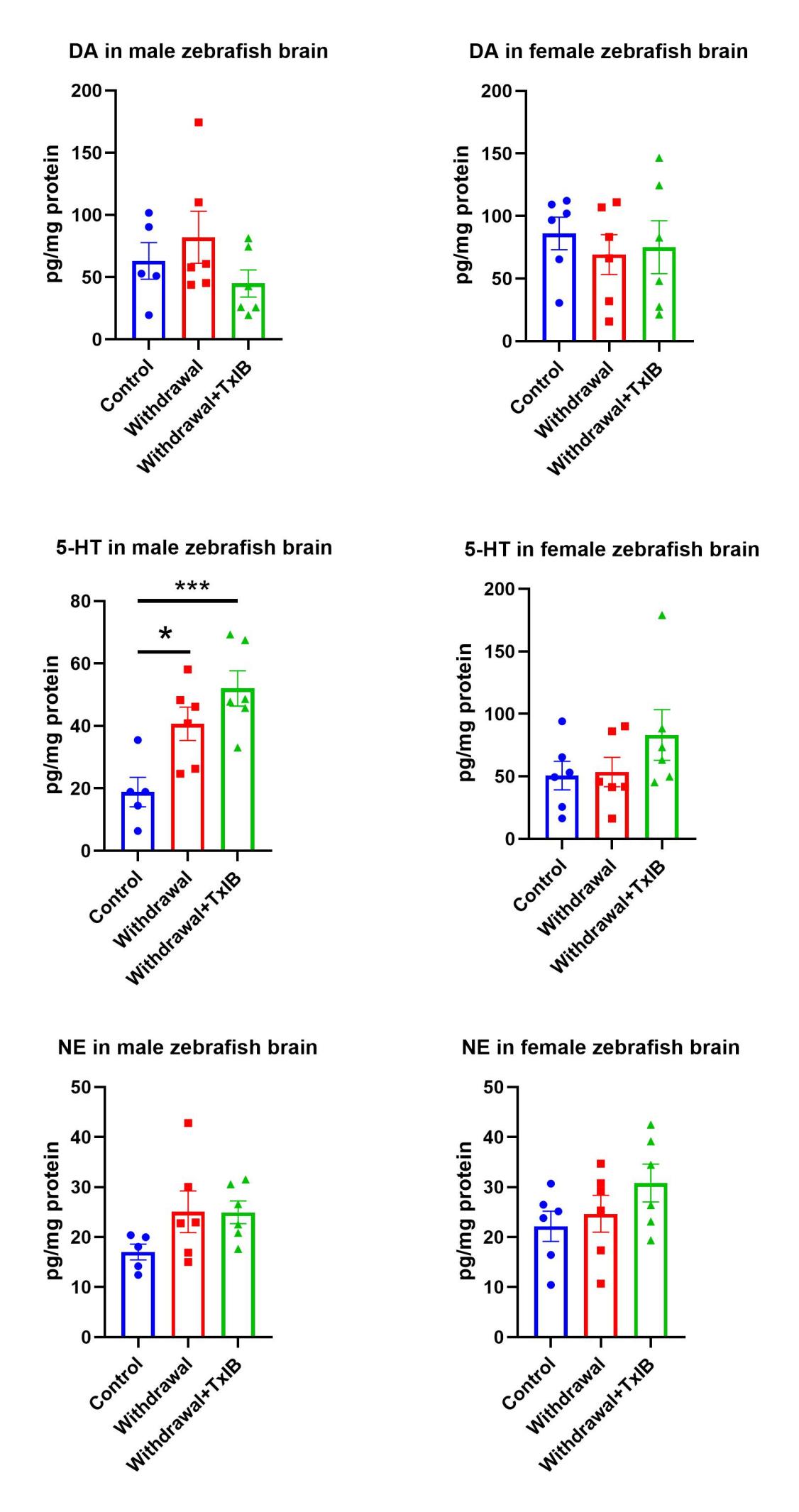


**Supplementary Figure 5**. Monoamine neurotransmitter content in the whole zebrafish brain of different genders. TxIB concentration of 1 mg/kg was used. Data are mean ± SEM of 6 zebrafish per group. * indicates *P* < 0.05, *** indicates *P* < 0.001 (one-way ANOVA followed by Tukey’s multiple comparisons test).

## Supplementary Tables

Supplementary Table 1. Functional roles of top 10 hub genes.

| No. | Gene symbol | Full name | Function |
| --- | --- | --- | --- |
| 1 | *ttnb* | titin, tandem duplicate 1 | Predicted to have ATP binding activity and protein kinase activity. Involved in myofibril assembly and skeletal muscle tissue development. Predicted to localize to plasma membrane. Is expressed in several structures, including cardiovascular system; floor plate; mesoderm; musculature system; and pectoral fin. Human ortholog(s) of this gene implicated in intrinsic cardiomyopathy (multiple) and myopathy (multiple). Orthologous to human TTN (titin). |
| 2 | *myl1* | myosin, light chain 1, alkali; skeletal, fast | Predicted to have calcium ion binding activity. Involved in skeletal muscle tissue development. Is expressed in adaxial cell; fin; musculature system; and somite. Orthologous to human MYL1 (myosin light chain 1). |
| 3 | *tnnc2* | troponin C type 2 (fast) | Predicted to have actin filament binding activity; calcium ion binding activity; and calcium-dependent protein binding activity. Involved in response to activity. Predicted to localize to troponin complex. Is expressed in fast muscle cell; head muscle; heart tube; skeletal muscle; and skeletal muscle cell. Orthologous to human TNNC2 (troponin C2, fast skeletal type). |
| 4 | *ttna* | titin, tandem duplicate 2 | A structural constituent of muscle. Involved in several processes, including heart contraction; myofibril assembly; and striated muscle tissue development. Localizes to centriole; cytoplasm; and nucleus. Is expressed in several structures, including cardiovascular system; mesoderm; musculature system; somite; and testis. Used to study atrial fibrillation and dilated cardiomyopathy. Human ortholog(s) of this gene implicated in intrinsic cardiomyopathy (multiple) and myopathy (multiple). Orthologous to human TTN (titin). |
| 5 | *actc1b* | actin alpha cardiac muscle 1b | Predicted to localize to dynactin complex. Is expressed in several structures, including EVL; mesoderm; musculature system; pericardial region; and trunk. Used to study nemaline myopathy. Human ortholog(s) of this gene implicated in atrial heart septal defect 5; dilated cardiomyopathy; dilated cardiomyopathy 1R; and hypertrophic cardiomyopathy 11. Orthologous to human ACTC1 (actin alpha cardiac muscle 1). |
| 6 | *mylpfa* | myosin light chain, phosphorylatable, fast skeletal muscle a | Predicted to have calcium ion binding activity. Is expressed in caudal fin; esophagus; musculature system; segmental plate; and somite. Human ortholog(s) of this gene implicated in distal arthrogryposis type 1c. Orthologous to human MYLPF (myosin light chain, phosphorylatable, fast skeletal muscle). |
| 7 | *tnnt3b* | troponin T type 3b (skeletal, fast) | Predicted to have tropomyosin binding activity; troponin C binding activity; and troponin I binding activity. Involved in sarcomere organization. Predicted to localize to troponin complex. Is expressed in musculature system and somite. Human ortholog(s) of this gene implicated in distal arthrogryposis type 2B and distal arthrogryposis type 2B2. Orthologous to human TNNT3 (troponin T3, fast skeletal type). |
| 8 | *actn3b* | actinin alpha 3b | Predicted to have actin binding activity and calcium ion binding activity. Is expressed in several structures, including musculature system; ovary; pharyngeal arch 3-7; post-vent region; and segmental plate. Orthologous to human ACTN3 (actinin alpha 3). |
| 9 | *mylz3* | myosin, light polypeptide 3, skeletal muscle | Predicted to have calcium ion binding activity. Is expressed in musculature system; pectoral fin; and somite. Orthologous to human MYL1 (myosin light chain 1). |
| 10 | *tnnt3a* | troponin T type 3a (skeletal, fast) | Predicted to have tropomyosin binding activity; troponin C binding activity; and troponin I binding activity. Involved in sarcomere organization. Predicted to localize to troponin complex. Is expressed in musculature system and somite. Human ortholog(s) of this gene implicated in distal arthrogryposis type 2B and distal arthrogryposis type 2B2. Orthologous to human TNNT3 (troponin T3, fast skeletal type). |
